# COVID-19 Lockdown and Social Capital Changes Among Youths in China

**DOI:** 10.34172/ijhpm.2021.17

**Published:** 2021-03-16

**Authors:** Miyang Luo, Dong Zhang, Pengyue Shen, Yun Yin, Shujuan Yang, Peng Jia

**Affiliations:** ^1^Xiangya School of Public Health, Central South University, Changsha, China.; ^2^International Institute of Spatial Lifecourse Epidemiology (ISLE), Hong Kong, China.; ^3^Department of Family and Preventive Medicine, College of Medicine, University of Arkansas for Medical Sciences, Little Rock, AR, USA.; ^4^West China School of Public Health and West China Fourth Hospital, Sichuan University, Chengdu, China.; ^5^Department of Land Surveying and Geo-Informatics, The Hong Kong Polytechnic University, Hong Kong, China.

**Keywords:** Social Science, Youth, Social Capital, COVID-19, Epidemic, Public Health

## Abstract

Social capital refers to the effective functioning of social groups through networks of relationships. The lockdown measures due to coronavirus disease 2019 (COVID-19) may change the social capital among youths. This study aimed to evaluate changes in social capital before and during COVID-19 lockdown among Chinese youths. It was based on the online COVID-19 Impact on Lifestyle Change Survey (COINLICS) conducted among 10 540 youths at three educational levels, including high/vocational school, undergraduate, and graduate, before and during COVID-19 lockdown. Measures of perceptions of social capital were adapted from a validated Chinese version of Health-related Social Capital Measurement based on youths’ characteristics of living and studying environment. Social capital was measured at four dimensions, including individual social capital (ISC), family social capital (FSC), community social capital (CSC), and society social capital (SSC). Overall, compared to before lockdown, ISC and CSC scores decreased, while FSC and SSC scores increased during lockdown. When stratified by educational levels, the trends for each dimension of social capital were consistent with the overall population. There were 43.9%, 5.7%, 32.1%, and 3.7% of the participants showing decreased scores during lockdown for ISC, FSC, CSC, and SSC, respectively, while 7.2%, 24.0%, 15.3%, and 10.7% of participants showed increased scores for ISC, FSC, CSC, and SSC, respectively. Our timely, large-scale study showed decreased social capital in individual and community dimensions and increased social capital in family and society dimensions during lockdown.

## Background

 Social capital is defined as the access to and use of resources embedded in one’s social networks,^1^ which describes trust, solidarity, cooperation, and reciprocity.^2-4^ The social networks and interpersonal relationships in social capital provide a useful framework for building a health-supporting and health-enhancing environment.^5^ Sufficient evidence suggested that social capital is associated with better physical and mental health, mediates the association between socioeconomic inequities and health,^6^ and serves as a protective factor against mortality.^7-9^ In addition, many studies have shown that social capital played an important role in crisis management processes,^10,11^ and may help to empower and mobilize the society and its members.^12^ In face of the coronavirus disease 2019 (COVID-19) pandemic, a recent study conducted in 84 countries found that social capital from civic engagement and confidence in state institutions were negatively associated with mortality, suggesting that public distrust for health authorities may influence the disease treatment and outcome.^13^

 To curb the spread of the pandemic, many countries have taken strict controlling measures, such as lockdown, which requires people to stay at home and reduce interactions with other people. In China, the lockdown was implemented in most provinces since February 2020 after the COVID-19 outbreak in January 2020.^14,15^ The lockdown measures may change the social capital among youths in at least three aspects. First, school closure was implemented for most high schools and universities, thus most students were living at home instead of living on campus. Second, face-to-face interactions with people not living together were greatly reduced owing to the social distancing and stay at home recommendations. Third, as people were very concerned about the pandemic, their emotions and feelings may be greatly influenced by pandemic-related information shared on various media platforms.^16,17^

 To evaluate the impact of social capital on lifestyle changes, we conducted a national survey that evaluated social capital in four dimensions, including individual, family, community, and society. We aimed to compare the differences in social capital between the month before the COVID-19 lockdown (January 2020, referred to as before lockdown) and the month during COVID-19 lockdown (February 2020, referred to as during lockdown) among youths in China. Our findings would provide empirical evidence for targeted interventions of social capital reconstruction among youths in China.

## Material and Methods

###  Study Design

 This is a cross-sectional study that used the COVID-19 Impact on Lifestyle Change Survey (COINLICS), a national retrospective online survey conducted in May 2020. A snowball sampling strategy was adopted to recruit the youths through chain-referral at three educational levels (ie, high/vocational school, college, or graduate) in China.^18,19^ The online questionnaire was distributed among several social media (WeChat and Tencent QQ) groups of educators, formed during the national conferences in the field (at least two educators in each province), to their eligible students. Next, all students who finished the questionnaire were encouraged to forward it to other students eligible.

###  Measures of Social Capital

 The questionnaire included questions on participants’ perceptions of social capital in the month before COVID-19 outbreak (January 2020) and during COVID-19 lockdown (February 2020). Measures of social capital were adapted from a validated Chinese version of Health-related Social Capital Measurement^20,21^ based on youths’ characteristics of living and studying environment. Participants’ level of social capital was assessed by 15 items in four dimensions, ie, the individual social capital (ISC), the family social capital (FSC), the community social capital (CSC), and the society social capital (SSC) (Supplementary file 1). The ISC measured individuals’ social network which was assessed using the following five items: (1) ‘you have many close contacts,’ (2) ‘you have many social interactions with people other than your family members,’ (3) ‘you always trust people who have social interaction with you,’ (4) ‘you always receive emotional/financial/instrumental support from friends/classmates,’ and (5) ‘you have a good relationship with your classmates.’ The FSC dimension was assessed using the following three items: (1) ‘you live with family members,’ (2) ‘you have a good relationship with your family (mainly including parents, brothers, and sisters),’ and (3) ‘you always receive emotional/financial/instrumental support from family members.’ The CSC dimension was assessed using five items, including: (1) ‘you frequently participate in activities organized by community organizations,’ (2) ‘you always receive support from community organizations,’ (3) ‘you always received emotional/financial/instrumental support from your teachers or instructors,’ (4) ‘you are very concerned about what happens in the same community/dormitory building,’ and (5) ‘you agree that people who live in the same community/dormitory can be trusted.’ The SSC dimension was assessed using two items, namely: (1) ‘you trust other health organizations/governmental organizations,’ and (2) ‘do you agree with the statement that talented people will be recognized by the society.’ The answer to each item ranges from 1 (strongly disagree) to 5 (strongly agree), with a higher total score indicating stronger levels of social capital.

###  Statistical Analyses

 The participants’ sociodemographic characteristics before lockdown and levels of social capital before and during lockdown were described as mean and standard deviation (SD) for continuous variables, and as percentage for categorical variables. Differences in these variables among youths of different educational levels were compared using t-test, analysis of variance, and Kruskal-Wallis test for continuous variables, or χ^2^ tests for categorical variables. Analysis of covariance was used to compare means of social capital scales before and during lockdown at three educational levels, with adjustment for age, sex, ethnicity, locality and province of residence. Statistical significance ​was declared if a two-sided *P* value was less than.05. R version 3.6.2 was used to perform all statistical analyses.

## Results

 A total of 10 540 participants aged 15 to 33 years old were included in the study. Of them, 2855 (27.1%) were high/vocational school students, 7419 (70.4%) were undergraduate students, and 266 (2.5%) were graduate school students. About 71% of the participants were female and 61.8% were from non-urban regions (Table 1).

**Table 1:** Baseline Characteristics of the Participating Youths

**Characteristics**	**Total ** **(n = 10540)**	**High/Vocational School Students (n = 2855)**	**Undergraduate Students ** **(n = 7419)**	**Graduate Students** ** (n = 266)**	***P*** ** Value**
Age (y)	19.9 ± 2.3	17.5 ± 1.2	20.6 ± 1.8	24.7 ± 3.4	<.001
Gender					<.001
Male	28.7	24.2	30.4	27.8	
Female	71.3	75.8	69.6	72.2	
Ethnicity					<.001
Han	94.9	96.7	94.4	91.0	
Minority	5.1	3.3	5.6	9.0	
Locality					<.001
Urban	38.2	24.3	42.6	62.8	
Non-urban	61.8	75.7	57.4	37.2	
Region					<.001
Northeast	0.3	0.1	0.3	3.4	
East	9.2	0.7	11.9	25.2	
West	87.1	99.2	83.5	56.8	
Central	3.4	0.0	4.3	14.6	

*Note*. Provinces, municipalities and autonomous regions include: Northeast (Liaoning, Jilin, Heilongjiang), East (Beijing, Tianjin, Hebei, Shanghai, Jiangsu, Zhejiang, Fujian, Shandong, Guangdong, Hainan), Central (Shanxi, Anhui, Jiangxi, Henan, Hubei, Hunan), and West of China (Inner Mongolia, Guangxi, Chongqing, Sichuan, Guizhou, Yunnan, Tibet, Shanxi, Gansu, Qinghai, Ningxia, Xinjiang).

 The changes in levels of social capital from before to during lockdown were shown in Table 2. Overall, we found that the adjusted mean social capital scores decreased slightly from 15.14 (SD = 0.64) to 14.01 (SD = 0.64) in individual dimension and from 13.36 (SD = 0.42) to 12.92 (SD = 0.42) in community dimension, and increased slightly from 12.70 (SD = 0.24) to 13.11 (SD = 0.24) in family dimension and from 7.11 (SD = 0.13) to 7.20 (SD = 0.13) in society dimension. The mean total social capital score decreased from 48.31 (SD = 1.11) to 47.24 (SD = 1.11). The trend of change was consistent across three educational levels after adjusting for age, sex, ethnicity, locality, and province of residence.

**Table 2:** Levels of Social Capital Among the Participating Youths Before and During the Lockdown Due to COVID-19

	**All (n = 10540)**		**High School Students** **(n = 2855)**		**Undergraduate Students** **(n = 7419)**		**Graduate Students** **(n = 266)**		***P*** ** Value**
	**Mean (SD)**	**Adjusted ** **Mean** ^c^ ** (SD)**	**Mean (SD)**	**Adjusted ** **Mean (SD)**	**Mean (SD)**	**Adjusted ** **Mean (SD)**	**Mean (SD)**	**Adjusted ** **Mean (SD)**	
ISC scale									
Before lockdown	15.14 (3.15)	15.14 (0.64)	14.30 (2.96)	14.61 (0.36)	15.43 (3.14)	15.31 (0.58)	14.30 (2.96)	14.61 (0.36)	<.001
During lockdown	14.01 (2.76)^a^	14.01 (0.64)^a^	13.27 (2.70)^a^	13.48 (0.36)^a^	14.27 (2.73)^a^	14.17 (0.58)^a^	13.27 (2.70)^a^	13.48 (0.36)^a^	<.001
FSC scale									
Before lockdown	12.70 (2.27)	12.70 (0.24)	12.69 (2.22)	12.62 (0.16)	12.73 (2.28)	12.73 (0.25)	12.69 (2.22)	12.62 (0.16)	<.001
During lockdown	13.11 (2.19)^a^	13.11 (0.24)^a^	12.83 (2.19)^b^	13.02 (0.16)^a^	13.21 (2.16)^a^	13.13 (0.25)^a^	12.83 (2.19)^b^	13.02 (0.16)^a^	<.001
CSC scale									
Before lockdown	13.36 (3.33)	13.36 (0.42)	12.44 (3.13)	13.00 (0.19)	13.71 (3.34)	13.47 (0.39)	12.44 (3.13)	13.00 (0.19)	<.001
During lockdown	12.92 (3.25)^a^	12.92 (0.42)^a^	12.07 (3.04)^a^	12.57 (0.19)^a^	13.25 (3.27)^a^	13.04 (0.39)^a^	12.07 (3.04)^a^	12.57 (0.19)^a^	<.001
SSC scale									
Before lockdown	7.11 (1.41)	7.11 (0.13)	7.30 (1.47)	7.17 (0.06)	7.04 (1.37)	7.09 (0.13)	7.30 (1.47)	7.17 (0.06)	<.001
During lockdown	7.20 (1.43)^a^	7.20 (0.13)^a^	7.38 (1.49)^b^	7.27 (0.06)^a^	7.14 (1.40)^a^	7.19 (0.13)^a^	7.38 (1.49)^b^	7.27 (0.06)^a^	<.001
Total social capital scale									
Before lockdown	48.31 (7.22)	48.31 (1.11)	46.73 (6.92)	47.40 (0.52)	48.92 (7.20)	48.60 (1.03)	46.73 (6.92)	47.40 (0.52)	<.001
During lockdown	47.24 (6.85)^a^	47.24 (1.11)^a^	45.55 (6.63)^a^	46.33 (0.52)^a^	47.87 (6.80)^a^	47.53 (1.03)^a^	45.55 (6.63)^a^	46.33 (0.52)^a^	<.001

Abbreviations: COVID-19, coronavirus disease 2019; ISC, individual social capital; FSC, family social capital; CSC, community social capital; SSC, society social capital. ^c^Adjusted for age, gender, ethnicity, locality, and province of residence. Values under a given variable were marked by letters a and b, if the difference before and during lockdown within a given educational level was significant (a^a^*P *< .001, ^b^*P *< .05). The differences of values among educational levels before COVID-19 and during the lockdown were estimated, with *P* value showed in the right-most column.

 The change in ISC was mostly due to the decrease in the number of close contacts, social interaction with people, and trust for people with social interaction (Table S1). The increase in FSC was mostly explained by the increase in the number of participants living with their family, friends, or classmates, while a decreased number of participants agreed that they had a good relationship with classmates. The decrease in CSC was supported by decreased activities, support, and trust in the community, while more participants reported their concern about what had happened in the community.

 When comparing social capital scores before and during lockdown on an individual basis, most participants showed stable social capital, with the percentage ranging from 48.9% to 85.6% across four dimensions (Table 3). Overall, 7.2% of the participants had increased ISC (mean change = 1.68, SD = 1.12) and 43.9% had decreased ISC (mean change = -2.87, SD = 1.86). The percentage of the participants with decreased ISC was highest for graduate students (57.9%), followed by undergraduate students (44.8%) and high school students (40.2%). In terms of FSC, 24.0% of the participants showed increased scores (mean change = 2.09, SD = 0.83) while 5.7% showing decreased scores (mean change = -1.67, SD = 1.14). The increase in FSC was more prominent among graduate students (54.5%) than the other educational levels. Additionally, 15.3% of the participants showed increased CSC (mean change = 1.79, SD = 1.20) and 32.1% of participants showed decreased CSC (mean change = -2.20, SD = 1.50), which was similar across the three educational levels. For SSC, 10.7% of the participants showed increased scores (mean change = 1.32, SD = 0.65), and 3.7% had decreased scores (mean change = -1.32, SD = 0.70). The total social capital score was stable among 32.8% of the participants, increased among 20.5% (mean change = 2.39, SD = 1.87) and decreased among 46.7% (mean change = -3.34, SD = 2.66).

**Table 3:** Changes in Social Capital Among the Participating Youths Before and During the Lockdown Due to COVID-19

	**All (n = 10540)**		**High School Students** **(n = 2855)**		**Undergraduate Students** **(n = 7419)**		**Graduate Students** **(n = 266)**		***P*** ** Value**
	**% Of Participants**	**Change in Score (Mean, SD)**	**% Of Participants**	**Change in Score (Mean, SD)**	**% Of Participants**	**Change in Score (Mean, SD)**	**% Of Participants**	**Change in Score (Mean, SD)**	
ISC scale									<.001
Increased	7.2	1.68 (1.12)	6.5	1.51 (1.12)	7.5	1.73 (1.11)	8.3	1.86 (1.17)	
Stable	48.9	-	53.2	-	47.7	-	33.8	-	
Decreased	43.9	-2.87 (1.86)	40.2	-2.80 (1.95)	44.8	-2.88 (1.84)	57.9	-3.06 (1.69)	
FSC scale									<.001
Increased	24.0	2.09 (0.83)	12.0	1.92 (0.90)	27.5	2.10 (0.81)	54.5	2.27 (0.85)	
Stable	70.3	-	82.7	-	66.6	-	41.0	-	
Decreased	5.7	-1.67 (1.14)	5.3	-1.82 (1.30)	5.9	-1.63 (1.08)	4.5	-1.42 (0.90)	
CSC scale									<.001
Increased	15.3	1.79 (1.20)	11.1	1.65 (1.11)	16.6	1.83 (1.21)	25.9	1.74 (1.31)	
Stable	52.5	-	63.7	-	48.4	-	47.4	-	
Decreased	32.1	-2.20 (1.50)	25.2	-2.23 (1.67)	35.0	-2.20 (1.46)	26.7	-1.86 (1.23)	
SSC scale									.011
Increased	10.7	1.32 (0.65)	10.1	1.25 (0.60)	11.0	1.34 (0.67)	10.2	1.11 (0.32)	
Stable	85.6	-	86.7	-	85.3	-	82.7	-	
Decreased	3.7	-1.32 (0.70)	3.2	-1.34 (0.65)	3.7	-1.33 (0.74)	7.1	-1.05 (0.23)	
Total social capital scale									<.001
Increased	20.5	2.39 (1.87)	15.0	2.11 (1.75)	22.2	2.45 (1.91)	33.8	2.60 (1.65)	
Stable	32.8	-	40.5	-	30.2	-	22.6	-	
Decreased	46.7	-3.34 (2.66)	44.5	-3.38 (2.98)	47.6	-3.34 (2.55)	43.6	-3.06 (2.00)	

Abbreviations: COVID-19, coronavirus disease 2019; ISC, individual social capital; FSC, family social capital; CSC, community social capital; SSC, society social capital. Difference in composition ratio among educational levels were estimated, with *P* value showed in the right-most column.

## Discussion

 In this study, we found that the total social capital score decreased during the lockdown period, and more specifically the social capital scores decreased on individual and community scales, while increasing on family and society scales. Although this change in social capital was consistent across three educational levels, most youths’ social capital during lockdown was stable compared to before lockdown. When comparing the change in social capital for each participant, more students showed a decreased social capital in individual and community dimensions, and a larger proportion of participants showed an increased social capital in family and society dimensions.

 The change social capital may be largely explained by the COVID-19 related lockdown measures. People were strongly recommended to stay at home and avoid face to face contact with others during the lockdown period. The proportion of close contacts and the social interactions with people other than family members reduced significantly, which might have resulted in decreased ISC. Moreover, most of the activities organized by communities were canceled during the lockdown and communications within communities were also decreased, which might lead to decreased CSC. It is also important to note that the trust for people with social interaction and people in the same community decreased, which may be as a result of the increased health concerns for social distancing. These findings were consistent with previous studies, which also reported a disruption of social contact and community participation after disasters or catastrophes, such as earthquakes.^10,11,22^ As social capital is closely linked with individual health status,^7^ interventions on social capital, especially in individual and community dimensions, may improve the general health status among youths. Moreover, previous studies reported the increased mental health issues among youths during COVID-19 lockdown,^23-25^ which suggested that online communication may not fully compensate for the emotional demands of face-to-face communication and community participation.^26^ It is therefore necessary to pay attention to mental health and promote interpersonal communications among youths during national disasters or crises.

 In this study, increased social capital was observed in family and society dimensions. The increased FSC may be explained by the increased connection with family as remote teaching was adopted for almost all educational institutions and students had more time to spend with their family during the lockdown. It is also important to note that the relationship with classmates slightly decreased among the students, which may be due to the decreased communications as students spent less time together outside of class time during the lockdown. The increased SSC reflected the increased trust for the government and health organizations in disease management and control and belief about equity in society.^27^ This increase of solidarity in society may help the nation to fight against the pandemic and get through the difficult times, which, in turn, may also increase the SSC among people.^28^

 This study has several limitations. First, social capital data before and during lockdown were self-reported, which may be subject to recall bias. However, even with recall biases, differences in participants’ answers before and during the lockdown should reflect their perceived changes in social capital in this unusual time period. Second, we only recruited student participants in this study, thus generalization of our results to other populations should be taken with caution, such as out-of-school youths. Also, students with more connections or friends may have a higher possibility of receiving the invitation and participating in the survey. Third, we could not conduct robust analysis on the basis of the limited numbers of samples from Hubei province, where the impact of lockdown policies may be different from that in other regions of China. Finally, other variables that might significantly affect the respondents’ social capital before and during the lockdown (eg, health status) should have been surveyed.

## Conclusion

 This timely, large-scale study revealed that, among the Chinese youths, social capital has increased in family and society dimensions and decreased in individual and community dimensions during the lockdown due to COVID-19. These findings serve as important evidence for necessity to continue rebuilding and improving youths’ social capital in all aspects during lockdown for epidemic prevention. For example, multiple stakeholders, including the governments, schools, and communities, should take actions to maintain or improve society-level trust (eg, updating epidemic information frequently), and promote communications between both communities and individuals in alternative ways (eg, updating what is happening in the community, organizing online activities for community members) during the unusual lockdown periods.

## Acknowledgement

 We thank the International Institute of Spatial Lifecourse Epidemiology (ISLE) and The Hong Kong Polytechnic University (1-BE58) for research support.

## Ethical issues

 The study was approved by the Sichuan University Medical Ethical Review Board (KS2020414). All subjects voluntarily participated in our study with informed consent, and the study was carried out in accordance with the Declaration of Helsinki (2013).

## Competing interests

 Authors declare that they have no competing interests.

## Authors’ contributions

 PJ and SY designed the study. ML led data analysis. All authors were involved in analyzing data and writing the manuscript, and approved the final version of the manuscript.

## Supplementary files

Supplementary file 1 contains Table S1.
