# Supplementary material for: COVID-19 Lockdown and Social Capital Changes Among Youths in China
Source: Int J Health Policy Manag. 2021 Mar 16;11(8):1301–6. doi: 10.34172/ijhpm.2021.17 (PMC9808357; doi:10.34172/ijhpm.2021.17)
Supplement: Supplementary file 1 — contains Table S1. [file ijhpm-11-1301-s001.pdf]

Article title: COVID-19 Lockdown and Social Capital Changes Among Youths in China

Journal name: International Journal of Health Policy and Management (IJHPM)

Authors' information: Miyang Luo[1,2]¶, Dong Zhang[3]¶, Pengyue Shen[4], Yun Yin[4], Shujuan Yang[4,2]*, Peng Jia[5,2]*

[1]Xiangya School of Public Health, Central South University, Changsha, China.

[2]International Institute of Spatial Lifecourse Epidemiology (ISLE), Hong Kong, China.

[3]Department of Family and Preventive Medicine, College of Medicine, University of Arkansas for Medical Sciences, Little Rock, AR, USA.

[4]West China School of Public Health and West China Fourth Hospital, Sichuan University, Chengdu, China.

[5]Department of Land Surveying and Geo-Informatics, The Hong Kong Polytechnic University, Hong Kong, China.

(*corresponding authors: rekiny@126.com & jiapengff@hotmail.com)

¶ Both authors contributed equally to this paper.

Supplementary file 1

Table S1. Participating youths' approval for items of the social capital before COVID-19 before and during lockdown

Variables		Grand total (n=10,540)	High/Vocational school students (n=2,855)	Undergraduate students (n=7,419)	Graduate students (n=266)
**Individual social capital scale (%, agree/strongly agree)**					
Q1	You have many close contacts				
	Pre-COVID-19	7.3	4.3	8.2	11.7
	Lockdown	1.4	0.8	1.6	3.0
Q2	You have many social interactions with people other than your family members				
	Pre-COVID-19	8.6	5.5	9.4	16.9

	Lockdown	3.9	2.4	4.6	3.4
Q3	You always trust people who have social interaction with you				
	Pre-COVID-19	60.4	49.9	64.1	70.3
	Lockdown	54.5	42.7	58.6	65.8
Q4	You always receive emotional/financial/instrumental support from friends/classmates				
	Pre-COVID-19	54.6	43.5	57.8	66.5
	Lockdown	53.8	42.8	57.5	65.8
Q5	You have a good relationship with your classmates				
	Pre-COVID-19	71.8	61.4	75.5	80.5
	Lockdown	68.9	57.9	72.8	77.8
**Family social capital scale (%, agree/strongly agree)**					
Q6	You live with family members				
	Pre-COVID-19	74.1	87.6	70.1	38.7
	Lockdown	93.9	94.3	93.9	90.6
Q7	You have a good relationship with your family (mainly including parents, brothers and sisters)				
	Pre-COVID-19	81.6	75.9	83.5	87.6
	Lockdown	80.0	75.1	81.7	86.1
Q8	You always receive emotional/financial/instrumental support from family members				
	Pre-COVID-19	73.5	64.2	76.9	79.0
	Lockdown	74.7	65.5	77.9	84.6
**Community social capital scale (%, agree/strongly agree)**					
Q9	You frequently participate in activities organized by community organizations				
	Pre-COVID-19	13.8	4.7	17.5	9.0

	Lockdown	7.2	2.7	9.0	8.3
Q10	You always receive support from community organizations				
	Pre-COVID-19	8.4	4.7	9.9	6.4
	Lockdown	7.6	4.3	8.9	6.4
Q11	You always receive emotional/financial/instrumental support from your teachers or instructors				
	Pre-COVID-19	26.0	20.9	27.8	30.8
	Lockdown	30.4	22.7	33.1	36.8
Q12	You are very concerned about what happens in the same community/dormitory building				
	Pre-COVID-19	36.5	33.7	37.3	45.1
	Lockdown	45.2	37.6	47.5	62.0
Q13	You agree that people who live in the same community/dormitory can be trusted				
	Pre-COVID-19	24.2	25.7	31.6	25.4
	Lockdown	22.9	24.7	32.0	24.4
**Society social capital scale (%, agree/strongly agree)**					
Q14	You trust other health organizations/governmental organizations very much				
	Pre-COVID-19	57.7	64.7	55.1	56.8
	Lockdown	60.8	67.25	58.3	59.8
Q15	Do you agree with the statement that talented people will be recognized by the society				
	Pre-COVID-19	46.9	46.4	46.8	54.5
	Lockdown	48.8	48.1	48.9	52.63
